# *Ror* homolog *nhr-23* is essential for both developmental clock and circadian clock in *C. elegans*

**DOI:** 10.1038/s42003-024-05894-3

**Published:** 2024-02-28

**Authors:** Shingo Hiroki, Hikari Yoshitane

**Affiliations:** 1 Tokyo Metropolitan Institute of Medical Sciences, Tokyo, Japan; 2 Department of Biological Sciences, School of Science, University of Tokyo, Tokyo, Japan

**Keywords:** Gene expression, Transcriptomics, Circadian rhythms

## Abstract

Animals have internal clocks that generate biological rhythms. In mammals, clock genes such as *Period* form the circadian clock to generate approximately 24-h biological rhythms. In *C. elegans*, the clock gene homologs constitute the “developmental clock”, which has an 8-h period during larval development to determine the timing of molting. Thus, the ancestral circadian clock has been believed to evolve into the oscillator with a shorter period in *C. elegans*. However, circadian rhythms have also been observed in adult *C. elegans*, albeit relatively weak. This prompts the question: if the clock gene homologs drive the developmental rhythm with 8-h period, which genes generate the circadian rhythms in *C. elegans*? In this study, we discovered that *nhr-23*, a homolog of the mammalian circadian clock gene *Ror*, is essential for circadian transcriptional rhythms in adult *C. elegans*. Interestingly, *nhr-23* was also known to be essential for the molting clock. The bilaterian ancestral circadian clock genes might have evolved to function over multiple periods depending on developmental contexts rather than a single 8-h period in *C. elegans*.

## Abstract (web summary)

A canonical circadian clock component in *C. elegans* was identified after decades of research: mammalian Ror homolog, nhr-23. It is essential for both the 8-h developmental rhythm and 24-h circadian rhythm.

## Introduction

Many organisms across phyla have internal clock systems which generate biological rhythms. These clocks operate on various timescales: e.g., the segmental clock induces a rhythmic somite segmentation every several hours^1,2^, and the circatidal clock generates the ~12.4-h period rhythm^3^.

One of the best-characterized biological clocks is the circadian clock. In mammals, the circadian rhythm is generated by a transcriptional network of genes known as clock genes, including transcriptional factors such as *Bmal1*, *Clock, Dbp*, and *Ror*^4^. Specifically, CLOCK and BMAL1 bind to a specific cis-element, E-box, to activate transcription of a series of genes including *Per1-3* genes, and the translated PER proteins inhibit the E-box-mediated transcription. Thus, these genes form a core negative feedback loop that generates the circadian oscillation^4^. Moreover, the core loop is interlocked with regulatory loops via D-box and RORE elements. D-box and RORE are activated by transcriptional factors DBP and ROR, respectively^5^. These three cis-elements generate specific phases of circadian transcription: E-box generates a morning expression, D-box generates a daytime expression, and RORE generates an evening expression^5–7^. Therefore, combinational regulation by these cis-elements generates the daily expression patterns of individual genes.

Interestingly, in the nematode *C. elegans*, the circadian clock gene homologs are considered to constitute the “developmental clock”. In *C. elegans*, molting occurs every 8–16 h depending on the temperature, and thousands of genes oscillate in synchrony with this molting cycle^8,9^. *C. elegans* homologs of the circadian clock genes are essential for this developmental rhythm with an 8-h period at 25 °C^8–11^. For instance, *lin-42*, the sole *Per* homolog, plays a critical role in determining the timing of molting during development. *lin-42* gene exhibits a clear developmental rhythm at the mRNA level^10^, and *lin-42* mutants exhibit an arhythmic molting cycle^9,12^. Furthermore, the *C. elegans Ror* homolog, *nhr-23*, controls rhythmic transcriptions of molting-related genes^13–16^ and is hence indispensable for developmental progression^15,17^. Thus, in *C. elegans*, it has been believed that the ancestral circadian clock had evolved into the developmental clock^8^.

While the clock gene homologs are used to generate the developmental rhythm with an 8-h period, circadian rhythms have also been observed in adult *C. elegans*^18–22^. Although much weaker than those in mammals, circadian transcriptional rhythms emerge after entrainment by temperature cycles in adult *C. elegans*^18^. However, in spite of the existence of such a transcriptome-wide circadian dataset published years ago, no study has identified canonical genes for circadian mRNA rhythm till now. Since the circadian transcriptome should reflect large information about the direct output from the core circadian clock, we assumed that the deeper analysis of the dataset leads to the identification of strong candidates for the core clock components.

In this study, we performed a reanalysis of the adult circadian transcriptome^18^ and larval developmental transcriptome^23^. We discovered that the expression of *Ror/nhr-23*-targeted genes exhibited adult circadian rhythms in a specific phase, while they exhibited developmental rhythms during larval development. We then showed that *Ror/nhr-23* is essential for the adult circadian rhythms, as well as for the larval developmental rhythms^13,17,24^. These results suggest that bilaterian ancestral circadian clock genes might have evolved to count multiple periods in C. *elegans*.

## Results

### The targets of clock gene homolog Ror/nhr-23 exhibited circadian transcriptional oscillations in adult *C. elegans*

**Fig. 1:**
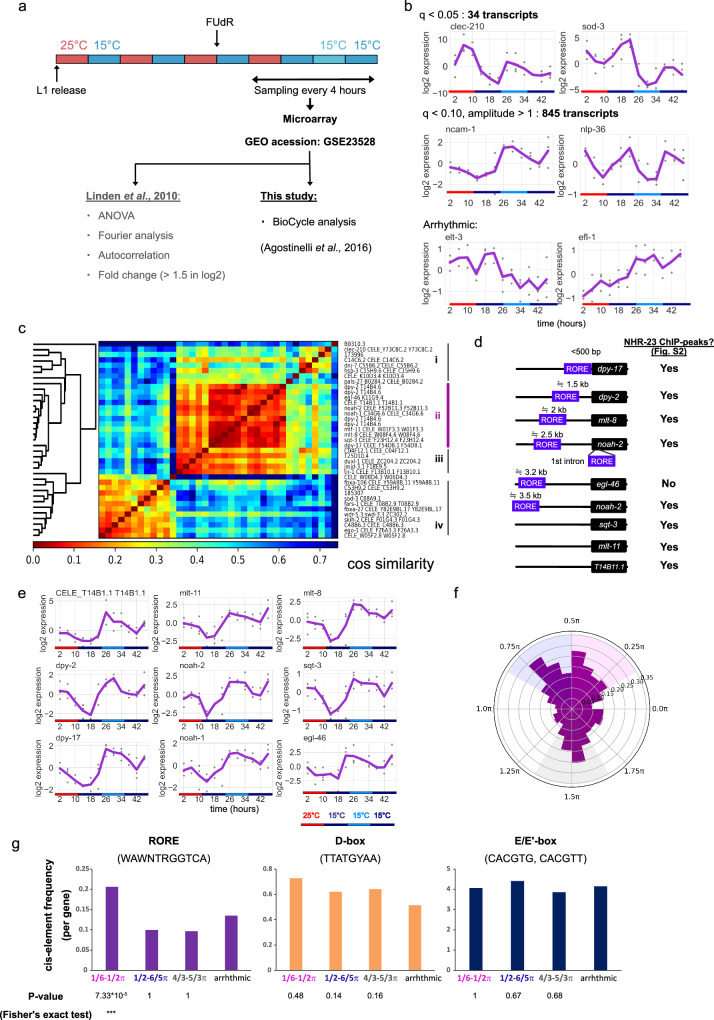
*Ror/nhr-23* and RORE cis-elements generate a specific phase of circadian transcription. **a** Reanalysis of the dataset from the previous study^18^. Briefly, worms were entrained by four 12-h warm (25 °C, red) and 12-h cold (15 °C, blue) temperature cycles (WC) and subsequently kept under the constant condition (CC) at 15 °C. Animals were sampled every 4 h over WC and CC. *n* = 3 biologically independent replicates at each time point. The dataset was reanalyzed using BioCycle in this study. **b** The result of BioCycle analysis. The number of periodic transcripts (arrays) in each criteria and representative gene expression were described. **c** Hierarchical clustering of *q* < 0.05 genes in BioCycle analysis. Magenta indicates specifically inter-correlated genes (“cluster ii). Colors indicate cosine distance between expression patterns. **d** The existence of RORE cis-elements and ChIP peaks in the clustered gene. **e** The expression pattern of the cluster ii genes. **f** Phase distribution of circadian rhythmic genes (*q* < 0.10 with 1< amplitude). Three peaks (magenta, blue, gray) were observed in the distribution. **g** Enrichment of cis-elements in gene regulatory region of the circadian rhythmic genes. *P* values were calculated by Fisher’s exact test followed by Holm’s correction. ***P < 0.001.

A previous study reported that the circadian rhythm emerges in *C. elegans* transcriptome after the entrainment by 12-h warm (25 °C) and 12-h cold (15 °C) temperature (WC) cycles (Fig. 1a)^18^. We reanalyzed this dataset to search for clues about the *C. elegans* circadian clock machinery. In the previous study, periodicity was calculated based on ANOVA, Fourier power, autocorrelation, and amplitude (fold change from minimum to maximum data point) (Fig. 1a). In this study, we used the BioCycle algorithm. BioCycle is a neural-net-based algorithm designed for circadian periodicity tests. Briefly, the neural network was trained to discriminate the periodic and aperiodic data, and then applied to the real dataset. Estimation of the oscillation parameters (amplitude, phase) is based on cosine fitting^25^. Here, we used BioCycle because it is reported to yield higher AUC compared to other commonly used algorithms such as JTK_CYCLE^26^, especially in cases of low signal-noise ratio^25^. Indeed, in our dataset, BioCycle detected a larger number of rhythmic genes compared to JTK_CYCLE(BioCycle-only: 615, JTK-only: 87, shared: 220, see Supplementary Fig. S1 and Supplemental Data 1 for details). Since these 615 genes involve genes with apparently clear rhythm such as *ncam-1*, *sod-3*, *mlt-8* (see Fig. 1b, e), we assumed that BioCycle exhibits a lower false negative rate in our dataset and is therefore suitable for our analysis. We analyzed whole time points (12 points every 4 h, during the WC temperature cycle and constant condition, CC), and calculated periodicity, phase, and amplitude for each gene (Supplemental Data 1). In addition, it should be noted that we found a set of arrays which were probably artifacts, which had exactly the same values as each other (Supplementary Fig. S2). However, these arrays could not pass any of our periodicity criteria below.

First, we focused on 34 transcripts with which met the criteria of *q* < 0.05 (Fig. 1b). Twelve of them were identified as rhythmic in the previous study^18^. To elucidate the temporal patterns of these genes, we performed hierarchical clustering after amplitude normalization. As a result, we observed 4 large clusters (Fig. 1c, “i–iv”). Remarkably, cluster ii consisted of genes with highly similar temporal patterns (Fig.1c, magenta). Indeed, the cluster ii genes exhibited a similar temporal expression pattern, i.e., oscillation in a specific phase (Fig. 1e). Hereafter, we call these genes “cluster ii genes”. Since these cluster ii genes were likely to be regulated by the same transcription factor, we investigated the detail of these genes and found that some of them (*dpy-2*, *noah-1*, *mlt-8*, *mlt-10,* *mlt-11*) were reported to be regulated by *nhr-23*^13–16,24^, the homolog of the clock gene *Ror*. To examine whether other cluster ii genes are also downstream of ROR/NHR-23, we explored the genome sequence and a previous comprehensive ChIP-seq dataset^27^. The dataset showed that most of the cluster ii genes indeed had NHR-23 ChIP peaks nearby their coding regions (Fig. 1d and Supplementary Fig. S3, 8 out of 9). Moreover, the ROR-regulated cis-elements, ROREs (WAWNTRGGTCA), were found in six of their regulatory regions (Fig. 1d). In addition, we investigated the homology of *nhr-23/Ror* among multiple species (Supplementary Fig. S4a, flies, zebrafish, mammals). Overall, the full amino acid sequences showed 30–40% similarity (36% when compared to *Mus musculus*). Specifically, the DNA-binding region (zinc finger) of homologs are highly conserved across species (78%, compared to *Mus musculus*). Therefore, *Ror/nhr-23* and ROREs are suggested to play canonical roles in generating the *C. elegans* circadian rhythm.

As mentioned earlier, mammalian ROREs generate a specific phase of circadian transcription^7^. To test if *C. elegans* ROREs are also enriched around the genes with the circadian rhythm in a specific phase, we first examined a phase distribution of the circadian expression. We extended the analysis to a broader set of genes (845 transcripts) with some false positives (*q* < 0.10, 1< amplitude). See Methods for the definition of amplitudes, where amplitude 1 in log2-tranformed dataset approximately corresponds to 2-fold change. This allowed us to describe the overall characteristics of the circadian genes, rather than focusing on an individual gene. There were 3 apparent peaks in the phase distribution (Fig. 1f): 1/6–1/2 π (magenta), 1/2–5/6 π (navy), and 4/3–5/3 π (gray). We then examined the enrichment of cis-elements in the regulatory region of genes with each of the peaks (4 kb upstream and introns). The frequency of ROREs around the genes in the “magenta” phase (1/6–1/2 π), the phase the cluster ii genes exhibited, was two times higher than those in other peaks and arrhythmic genes. However, we did not find any evidence for the enrichment of other circadian-related cis-elements, D-box and E/E’-box, in any of the peaks (Fig. 1g). In addition, we explored the rhythmicity of *C. elegans* gene homologs in mice based on RhythmicDB^28^ (Supplementary Fig. S4b). As a result, we found that 35% of the rhythmic genes in *C. elegans* also exhibit circadian rhythms in mice. This percentage is slightly higher compared to the overall proportion of rhythmic genes among all mouse genes that have *C. elegans* homologs (28%, *P* = 0.067 in Fisher’s test). However, given the limited overlap, we could not draw a definitive conclusion from this analysis.

These results suggest that genes with RORE are activated by ROR/NHR-23 at the specific time of the day in *C. elegans* as reported in mammals, while other, perhaps unknown, cis-elements might be responsible for the generation of other circadian phases.

### *Ror/nhr-23* generates developmental rhythm with 8-h period during larval stage

While our results suggest a putative contribution of *Ror/nhr-23* to *C. elegans* circadian clock, *Ror/nhr-23* has been considered to be a core developmental clock gene of the molting cycle, which oscillates with a shorter period (~8 h)^13,15,24^. In *C. elegans*, the homolog of the other mammalian RORE-binding clock gene, *Rev-erb/nhr-85*, does not show developmental rhythm, and loss of *nhr-85* marginally impairs development in *C. elegans*^29^. Therefore, *Ror/nhr-23* has a major contribution to RORE-dependent developmental rhythm in *C. elegans*.

**Fig. 2:**
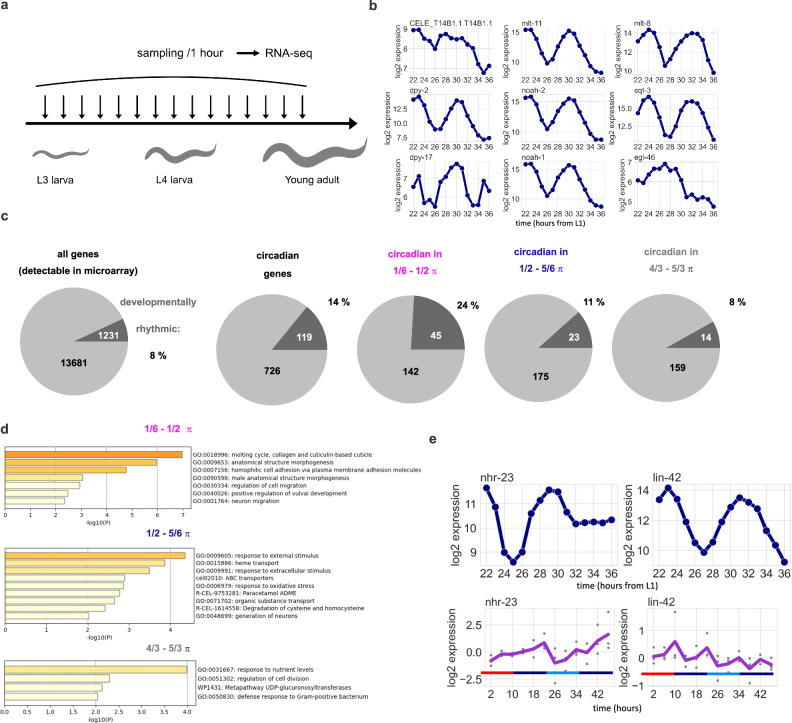
RORE-targeted circadian genes oscillate with 8 h period during larval development. **a** Summary of the dataset for developmental transcription. Animals from 22 to 36 h after the L1 stage (L3 to young adults) were sampled every 1 h. *n* = 1 for each time point. **b** Expression of RORE-targeted genes (“clustered gene” in Fig. 1) during larval development. **c** The proportion of developmentally rhythmic genes in circadian rhythmic genes (*q* < 0.10 with 1< amplitude). **d** The result of Metascape analysis^30^ in genes oscillating in circadian rhythm with a specific phase. **e** Expression of *Per/lin-42* and *Ror/nhr-23* during larval development and adulthood.

To examine whether *nhr-23* downstream genes indeed show the 8-h rhythm during larval development, we reanalyzed the time series RNA-seq dataset from L3 to young adult^23^ (Fig. 2a). First, we focused on the cluster ii genes in Fig. 1c and found that most of them indeed exhibit 8-h expression rhythms (Fig. 2b, 7 out of 9). Subsequently, we expanded our analysis to encompass a broader spectrum of genes to explore the extent of overall overlap between circadian rhythmic genes and developmentally rhythmic genes. We applied BioCycle to detect periodicity in 8–10 h, and thus defined the “developmentally rhythmic” genes with the same criteria as those used in the circadian periodicity test, i.e., *q* < 0.10 and 1< amplitude. With this test, 6% of all genes were detected as developmentally rhythmic (Fig. 2c and Supplemental Data 2), while 14% of circadian rhythmic genes showed developmentally rhythmic expression, suggesting that the output from the circadian clock partly, if not largely, overlaps with that from the developmental clock. Notably, among them, genes with the RORE-regulated phase (1/6–1/2 π) showed a remarkable overlap (24%) with developmentally rhythmic genes (Fig. 2c). Likewise, when we applied “annotation-based enrichment analysis (Metascape)^30^ to genes oscillating in each of the phases, only the phase 1/6–1/2 π showed enrichment of the genes related to molting cycles (Fig. 2d). These results imply that the circadian clock partially shares the clock components with the developmental clock, especially in terms of *Ror/nhr-23* and RORE-dependent system.

In addition, consistent with the previous reports^10,18^, mRNA of the canonical developmental clock gene, *Per/lin-42*, showed a developmental rhythm but not a circadian rhythm (Fig. 2e), suggesting that the developmental clock is somewhat different from the circadian clock. *nhr-23* also shows arrhythmic expression in adulthood (Fig. 2e), but this is probably due to its high germline expression for spermatogenesis after sexual maturation^31^.

Taken together, though the outcome of the circadian clock does not completely overlap with that of the developmental clock, the functional rhythm of *Ror/nhr-23* plays a significant role in generating gene expression rhythms in both contexts.

### Ror/nhr-23 is a critical regulator not for only the larval molting rhythm but also for the adult circadian rhythm

**Fig. 3:**
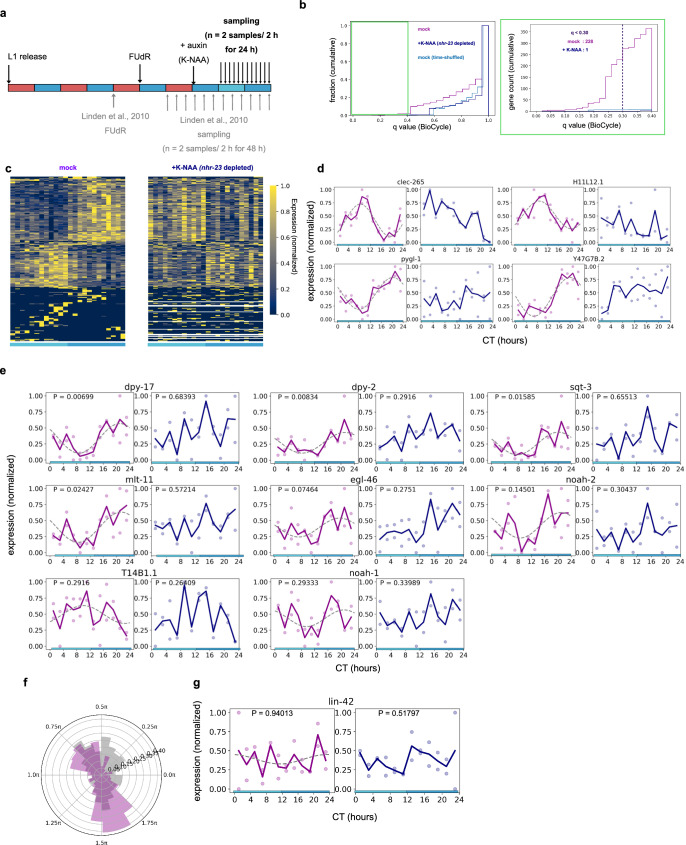
*Ror/nhr-23* is essential for circadian transcription in *C. elegans*. **a** Summary of the experiments for RNA-seq. The entrainment schedule was based on the previous study. The test group was treated with auxin (K-NAA) to deplete NHR-23 12 h before sampling was started. Animals were sampled every 2 h for a day in the constant condition at 15 °C. *n* = 2 biologically independent replicates. **b** Cumulative histogram of the q value calculated by BioCycle in circadian transcription for all genes *q* value in *q* < 0.40 gene count. Apparently, more genes are rhythmic in the mock dataset, compared to the auxin-treated dataset. For reference, the number of *q* < 0.30 genes were indicated in the graph area. **c** Expression pattern of rhythmic genes (*q* < 0.30 in mock condition). The gene expressions were normalized so that maximum = 1 and min = 0. Auxin treatment eliminates overt rhythms. rep. stands for biological replicates. **d** Representative plots of raw TPM values of circadian rhythmic genes. *P* indicates uncorrected *P* values in BioCycle analysis. Colors indicate the experimental group (magenta: mock, +K-NAA: navy). **e** Expression of the RORE-targeted genes (cluster ii genes in Fig. 1) in the datasets. Colors indicate the experimental group as (**d**). **f** Phase distribution of *q* < 0.30 genes in the mock condition (magenta). The gray histogram shows the phase distribution of Fig. 1f. **g** Arrhythmic expression pattern of *Per/lin-42* in our RNA-seq dataset.

To directly test the contribution of *Ror/nhr-23* to the adult circadian rhythm, we depleted NHR-23 specifically in adulthood using the Auxin-Induced Degron (AID) system^32,33^. We entrained animals by the same temperature cycles as the previous report^18^ and then sampled worms every 2 h for RNA-seq analysis (Fig. 3a). By adding the auxin after 84 hours NHR-23 is depleted after the final molt, thereby effect to molting is avoided. Considering that the acute NHR-23 depletion would substantially affect the temporal component, we took 12 h after the treatment to avoid such an acute effect. Therefore, we only sampled worms under CC conditions. We did not observe any apparent abnormality or behavioral defect, such as nose touch response.

In the resulting dataset (Supplemental Data 3), we could detect only a small number of circadian rhythmic genes in the mock condition, compared with the previous study^18^: only 228 genes met the criteria of *q* < 0.30 by BioCycle analysis in the mock condition (Fig. 3b). However, the number of rhythmic genes based on *q* values was still substantially larger than that in the time-shuffled dataset (Fig. 3b, d). Therefore, rhythmic components are considered to be indeed embedded in our dataset. The phase distribution of rhythmic (*q* < 0.30) genes are largely consistent with the previous study, though the RORE-enriched phase (1/2–1/6 π) was not obvious in *q* < 0.30 (Fig. 3f). Some of the RORE-targeted genes were detected as circadian rhythmic at least at uncorrected *P* value *P* < 0.05, but not all of them (Fig. 3e). These differences from the previous study might be due to the limited sampling time (24 h in this study vs. 48 h in the previous study) or the difference in the strains (degron knock-in strains vs. N2).

However, even with the low detection power, the effect of *nhr-23* knockdown was evident: treatment with the auxin analog K-NAA^34^ decreased the circadian periodicity of gene expression to the level of that in the time-shuffled dataset (Fig. 3b). Only one gene was detected as periodic in the *nhr-23* knockdown dataset even in *q* < 0.30, and most *q* < 0.30 genes in the mock dataset lost overt rhythm by auxin treatment (Fig. 3c, d and Supplementary Fig. S5). The abolishment of circadian rhythms is not limited to a specific phase (Fig. 3c, e), suggesting that *nhr-23* is involved in a canonical circadian oscillator in adult *C. elegans*, rather than merely generating a specific phase of circadian transcription. Overall, our findings indicate that *nhr-23/Ror* is one of the core circadian clock genes in *C. elegans*.

## Discussion

In this study, we discovered that *Ror/nhr-23* is not only an essential clock gene for developmental rhythm (with 8 h period in 25 °C) as previously reported^16,17,24^, but also a core circadian clock gene of adult *C. elegans*. Genes with RORE exhibited circadian transcription in a specific phase, and depletion of *Ror/nhr-23* in adulthood abolished the circadian rhythm itself. So far, no study has identified a canonical circadian clock gene in *C. elegans*, or the common component of the developmental and circadian clocks. The *Ror/nhr-23* is involved in the core transcriptional network of the developmental clock during larval stages, and may come to be involved in that of the circadian clock after the completion of larval development.

Even in the light of our data, however, the clockwork of *C. elegans* has still been enigmatic. Moreover, we have no idea of how the transition between these two clockworks occurs. To our knowledge, even in the developmental clock, no study has clearly demonstrated how the clock gene homologs interact to generate the 8-h oscillation. One interesting observation is that *Per/lin-42* mRNA does not show a circadian rhythm^18^ (Figs. 2e and 3g). Given this, *Ror/nhr-23* might be involved in the *Per/lin-42*-based oscillatory system during larval development. After the completion of larval development, the contribution of *lin-42* is somehow decreased, and another oscillatory system might emerge.

Circadian rhythms exist across diverse species, while the core clock components differ between distant species. For example, FRQ and WC in *Neurospora*^35^ and TOC1 and CCA in plants^36^ form transcriptional feedback loops. However, some molecular components, such as CK1^37^, calcium^38^, and redox oscillations^39^, are conserved even in organisms with different clock genes, suggesting the possibility that these elements may exist prior to the transcriptional feedback loops. Intriguingly, the redox oscillation was reported in *C. elegans*^40^. Considering these findings, while the homologous oscillators of the mammalian circadian clock have evolved to the developmental clock in *C. elegans*, it is possible that these conserved components drive functional oscillation of *nhr-23* after the developmental clock, resulting in a circadian functional rhythm of *nhr-23*.

To date, the “ancestral clock” of the circadian clock was thought to have evolved to generate the 8-h developmental rhythm in *C. elegans*. However, our results indicate that the core component of *C. elegans* developmental clock still possesses an ability to generate circadian rhythms. That is, the ancestral clock genes evolved to flexibly generate the biological rhythms with multiple timescales according to the context, i.e., molting during larval development and daily cycles in adulthood. While decades of research have revealed how robustly the circadian clocks are tuned to 24-h, few studies have focused on how flexible the clock gene network could be. Our findings provide deeper insights into the evolution of the biological clock system.

## Methods

### *C. elegans* strains and culture

Animals were maintained on nematode growth media (NGM)^41^. Wild-type animals correspond to the Bristol strain N2. For NHR-23 knockdown experiments, we used JDW259: *wrdSi55[eft-3p:TIR1:F2A:mTagBFP2:tbb-2 3*′UTR, I:-5.32]; nhr-23(kry61(nhr-23::AID*::TEV::3xFLAG)).

### Analysis of transcriptome dataset

The dataset was acquired from GEO (circadian transcriptome^18^: GSE23528, developmental transcriptome^23^: GSE52910). For the circadian transcriptome analysis. we used the temperature cycle and constant temperature (WC-CC) dataset. For developmental transcriptome analysis, we used “N2 untreated” dataset.

We used BioCycle R script^25^ for periodicity tests and estimation of phases and amplitudes: For circadian transcriptome, a range of the period was set to 20–28 h. For developmental transcriptome, the range was set to 8–10 h. We used BH method to calculate *q* values. In the dataset, we found a substantial number of arrays with identical values, which we suspect to be artifacts. However, these arrays were not included in our analysis. In BioCycle analysis, the amplitude is defined as variance of the signal to the variance of a cosine signal. We used JTK_CYCLE using R script for comparison in Supplementary Fig. S1.

For cis-element analysis, we acquired genomic positions and introns and upstream regions of genes from WormBase (www.wormbase.org) using Python web service API. Then, the sequence was extracted from WBcel235 genome assembly. All introns and 4 kb upstream from the first exons were searched for enrichment of cis-elements. The number of cis-elements in each regulatory region is described in Supplemental Data 3.

All statistical analyses were performed with Python3 scripts.

The result of reanalysis by BioCycle is described in Supplemental Data 1 and 2.

### RNA extraction and sequencing

In total, 1000–2000 L1-synchronized animals were cultivated on 10-cm dishes under temperature cycles (25 °C : 12 h, 15 °C : 12 h) as described in Fig. 3a. After 48 h (L4 stage), animals were transferred to other 10-cm plates with 10 µM FUdR to eliminate offsprings. Then, after 36 h (84 h post L1 release), animals were transferred to plates with 400 mM K-NAA.

From 97 to 119 h post L1 release, animals were sampled every 2 h: Animals were collected from plates and washed at least 3 times using cold wash buffer. Animals were then frozen in liquid nitrogen. All manipulations were done in at most <15 min.

RNA was extracted from the samples with TRIzol (Invitrogen) and RNAeasy mini kit (Qiagen). All RNA samples showed RIN > 9. Strand-specific RNA-Seq libraries were prepared by BGI (China) and sequenced on the DNB-Seq platform (150 bp pair end, 12,000,000 reads). Quantification of gene expressions was performed by Salmon^42^ with *C. elegans* mRNA sequences (WS284 version). The dataset after BioCycle analysis is described in Supplemental Data 3.

### Statistics and reproducibility

We treated animals derived from individual culture plates as biologically independent replicates. We used 36, 16, 24 samples in Figs. 1–3 (3 replicates from 12 time points, 1 replicate from 16 time points, 2 replicates from 12 time), respectively. Statistical tests for periodicity are based on BioCycle. We used BH-corrected *P* value (*q* value) to control False Discovery Rate (FDR) in multiple comparison. For cis-element enrichment analysis, Fisher’s test was performed to the binarized value (each gene regulatory region has/does not have cis-element), then corrected by Holm’s correction.

### Reporting summary

Further information on research design is available in the Nature Portfolio Reporting Summary linked to this article.

### Supplementary information

Supplementary Information

Description of Additional Supplementary Files

Supplemental Data 1

Supplemental Data 2

Supplemental Data 3

Supplemental Data 4

Supplemental Data 5

Reporting Summary

## Data Availability

Our RNA-seq data can be accessed in GEO (Accession: GSE233891). The dataset used for reanalysis is accessible as circadian transcriptome: GSE23528, developmental transcriptome^3^: GSE52910). The result of the analysis is available as Supplemental Data 1–4. Source Data is available as Supplemental Data 5.

## Code availability

All codes used in this study will be provided upon the request to corresponding authors.
