# Supplementary material for: Ror homolog nhr-23 is essential for both developmental clock and circadian clock in C. elegans
Source: Commun Biol. 2024 Feb 28;7:243. doi: 10.1038/s42003-024-05894-3 (PMC10902330; doi:10.1038/s42003-024-05894-3)

Supplemental Information

Fig.S1.

Comparison between BioCycle and JTK_CYCLE.

The amplitude of the cosine fit was calculated, and filtered by amplitude > 1 to cut off the artifacts. Then we compared genes with a threshold of q < 0.1 or q < 0.2 in each of the algorithms. For details, see Supplemental Data 1.

Fig. S2

Putative artifacts in the dataset.

(A) (left) Histogram of estimated amplitudes in all genes and (right) zoomed graph around amplitude = 0.00. An abnormally high peak is observed around amplitude = 0.0472.(B) (left) Histogram of estimated amplitudes in all genes and (right) zoomed graph around p = 0.02 . An abnormally high peak is observed around amplitude = 0.0197. (C) Example plot of gene expression included in the abnormal peaks. Genes show identical expression patterns. The complete list of putative artifacts is described in **Supplemental Data 4**.

Fig. S3

ChIP peaks around the cluster ii genes.

ChIP results acquired from www.wormbase.org, which is based on Araya et al., 2014.

Fig.S4

Alignment of ROR/NHR-23 homologous proteins.

(a)The full-length amino acid sequences of ROR/NHR-23 homologous proteins were aligned using ClustalW. The following proteins were used as the homologs in each species: NHR-23: *Caenorhabditis elegans*, dHR3: *Drosophila melanogaster*, RORAA: *Danio rerio*, RORG: *Mus Musculus*. (b) Number of mice(*Mus Musculus*) homologs with circadian rhythms. Rhythmic mice genes were extracted from Rhythmic DB (with q < 0.05). The definition of homologs is based on “homology” information in WormBase.

Fig. S5

RNA-seq results with the raw TPM value.

Plot with the TPM value before normalization. Genes and color codes were same as those in Fig.3D, E, G.

Fig. S1

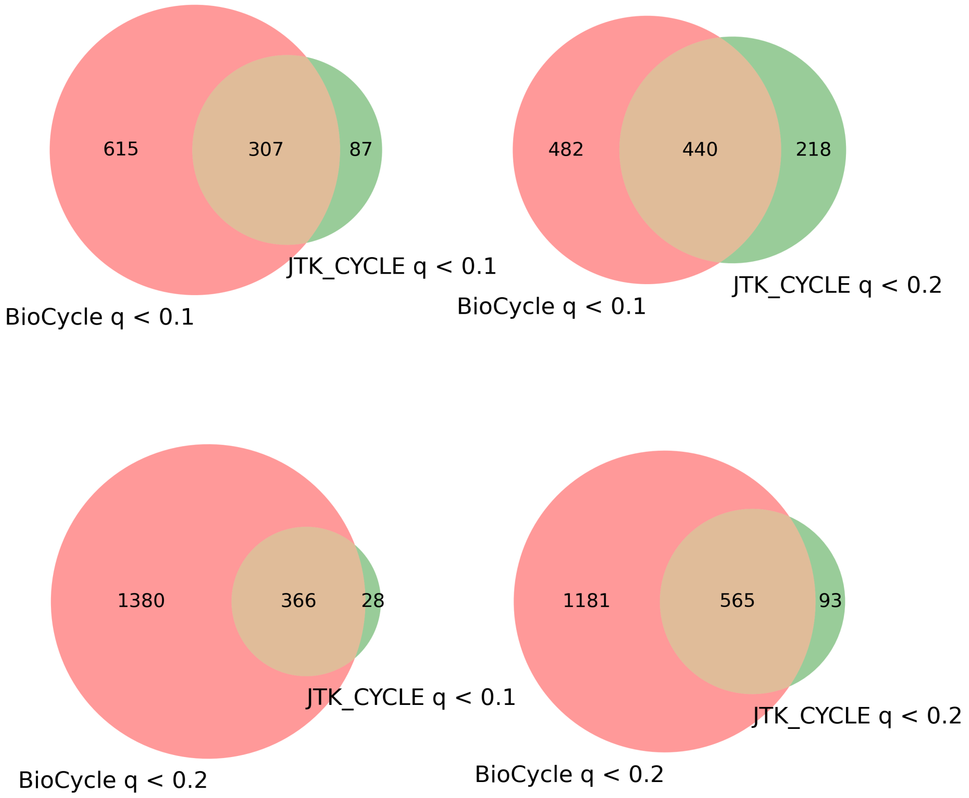

615
307
87
JTK_CYCLE q < 0.1
BioCycle q < 0.1
482
440
218
BioCycle q < 0.1
JTK_CYCLE q < 0.2
1380
366
28
JTK_CYCLE q < 0.1
BioCycle q < 0.2
1181
565
93
JTK_CYCLE q < 0.2
BioCycle q < 0.2

Fig. S2

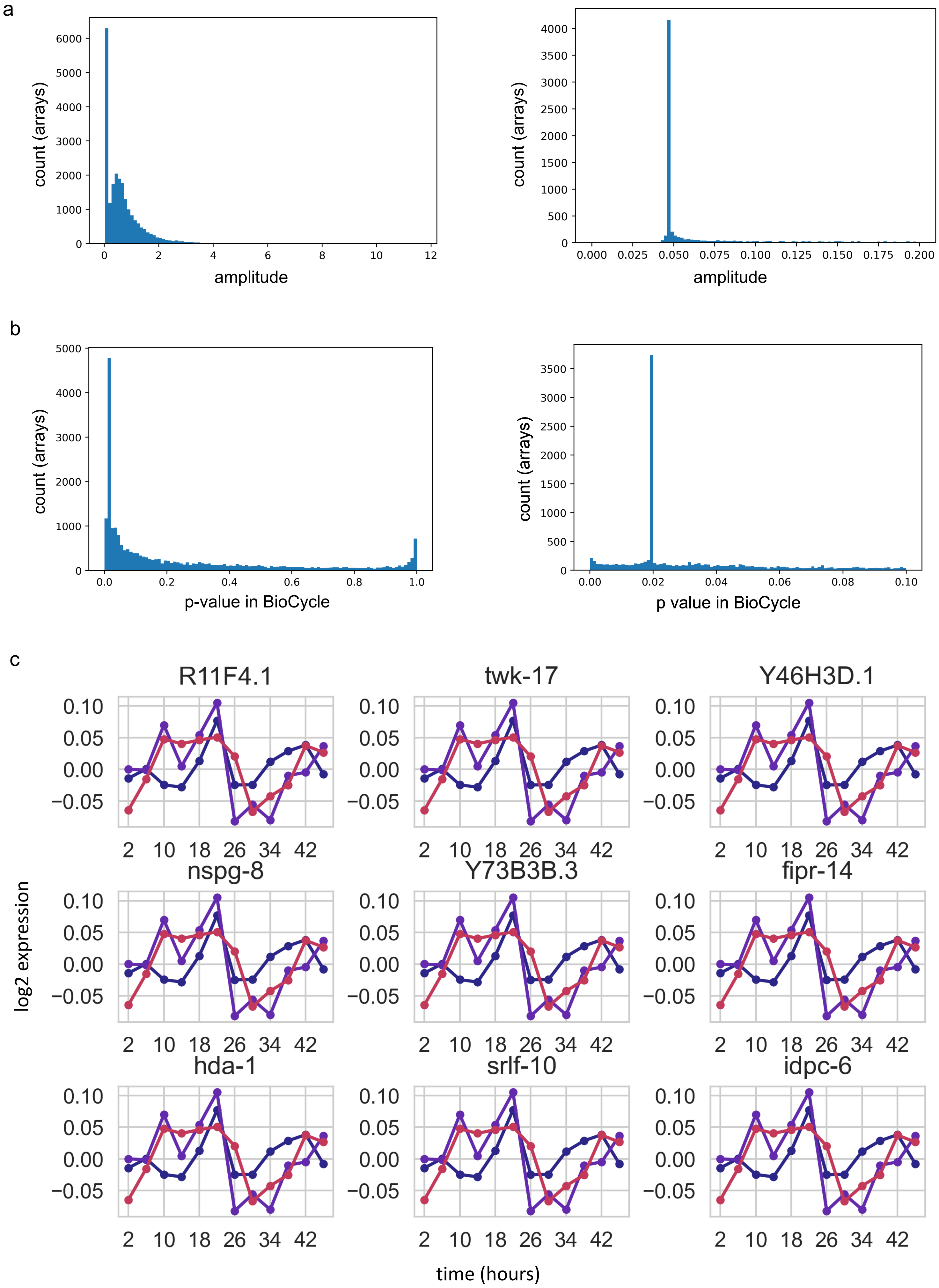
a
count (arrays)
amplitude
count (arrays)
amplitude
b
count (arrays)
p-value in BioCycle
count (arrays)
p value in BioCycle
c
R11F4.1
twk-17
Y46H3D.1
nspg-8
Y73B3B.3
fipr-14
hda-1
srlf-10
idpc-6
log2 expression
time (hours)

Fig.S3

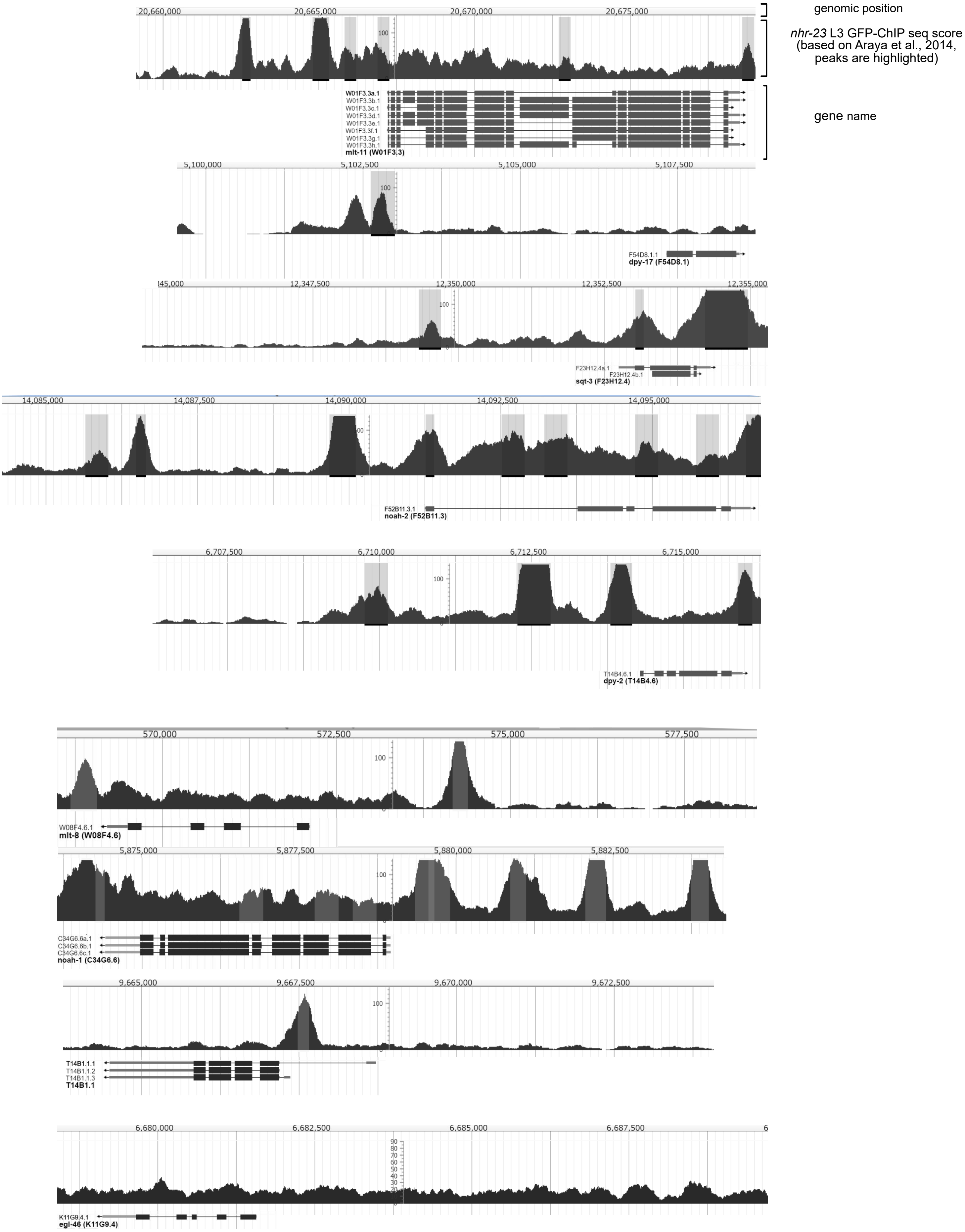
genomic position
nhr-23 L3 GFP-ChIP seq score (based on Araya et al., 2014, peaks are highlighted)
gene name
mlt-11 (W01F3.3)
dpy-17 (F54D8.1)
sqt-3 (F23H12.4)
noah-2 (F52B11.3)
dpy-2 (T14B4.6)
mlt-8 (W08F4.6)
noah-1 (C34G6.6)
T14B1.1
egl-46 (K11G9.4)

Fig. S4

a

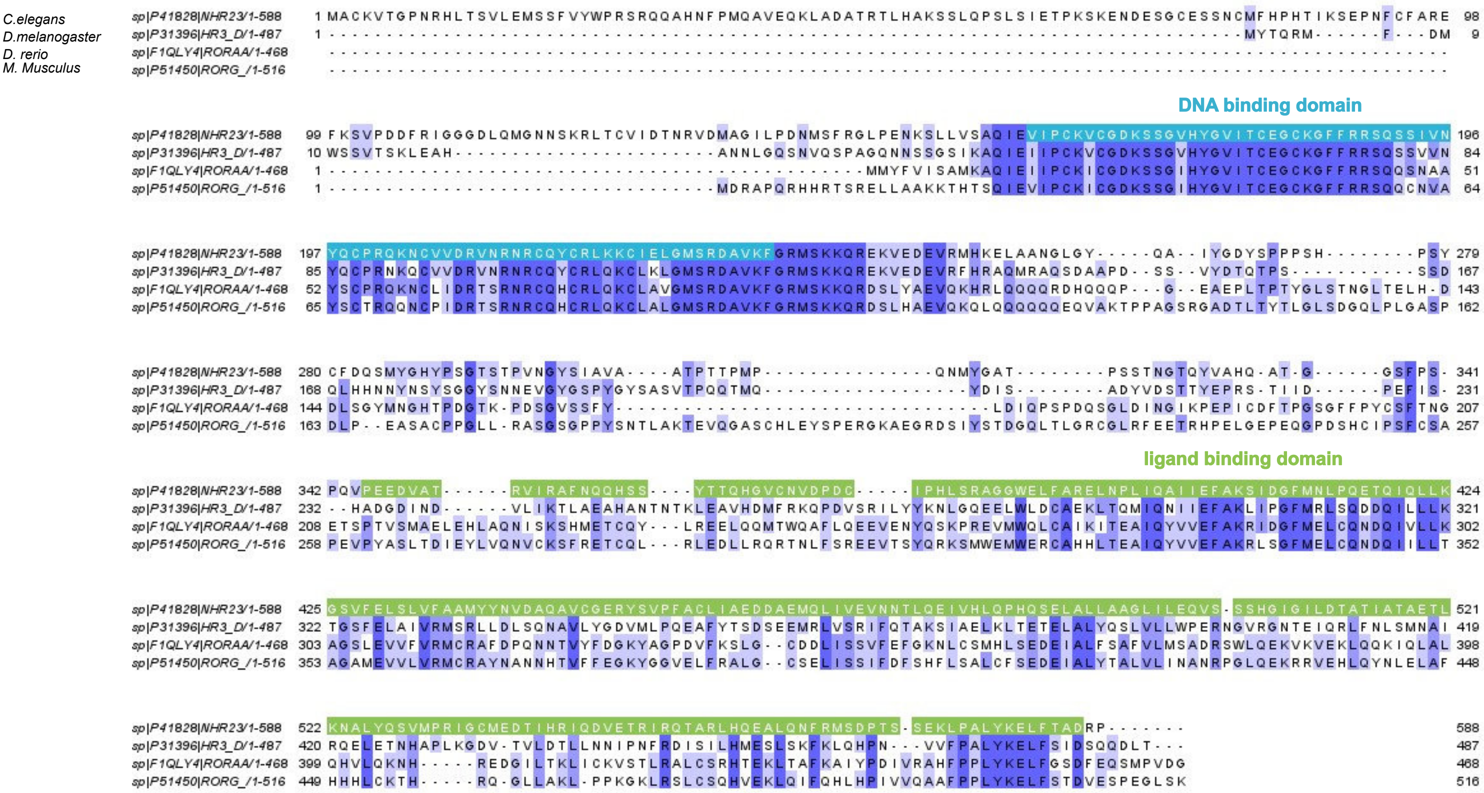

b

	arrhythmic in mice homolog	rhythmic in mice homolog
arrhythmic in worm homolog	3057	1178
rhythmic in worm homolog	89	48

Fig.S5

For 3d

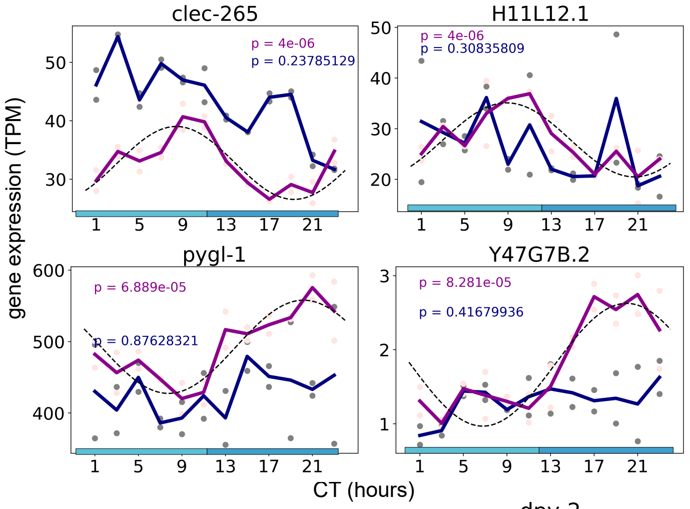

For 3e

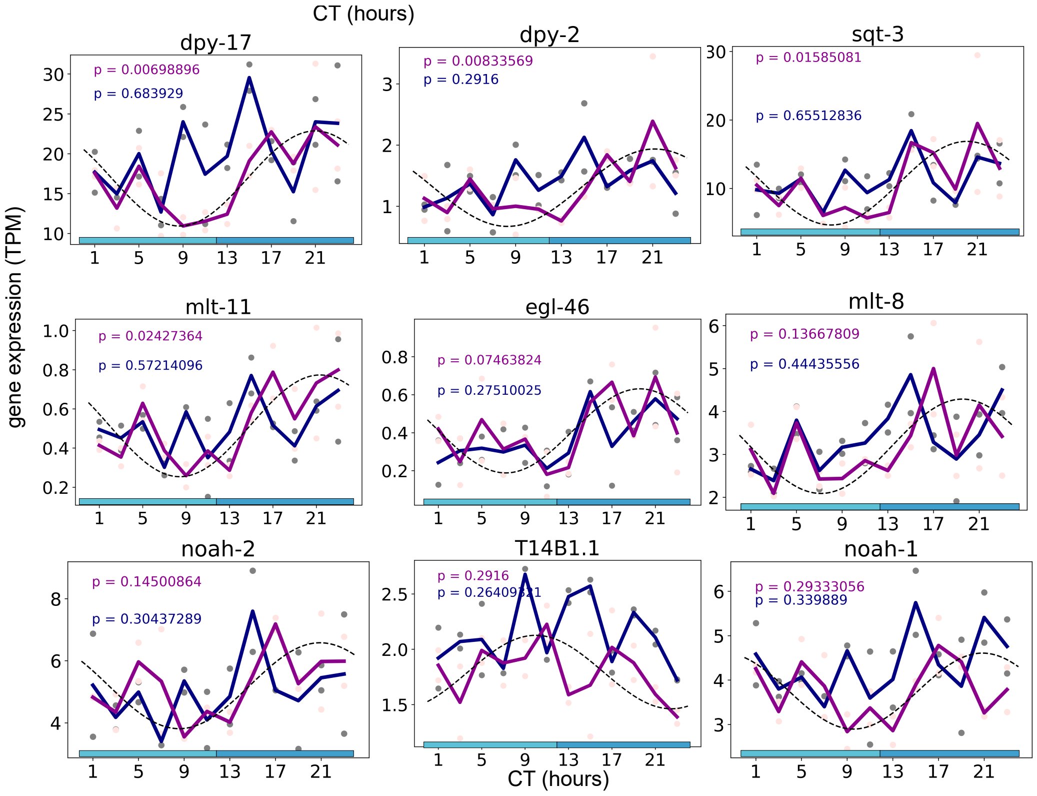

For 3g

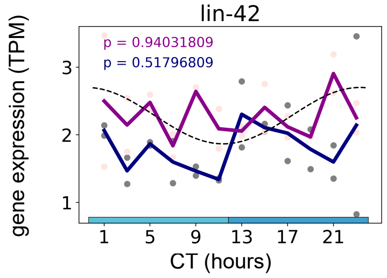

Supplement: Supplementary file 1 — Supplementary Information [file 42003_2024_5894_MOESM1_ESM.pdf]
