# Supplementary material for: Ror homolog nhr-23 is essential for both developmental clock and circadian clock in C. elegans
Source: Commun Biol. 2024 Feb 28;7:243. doi: 10.1038/s42003-024-05894-3 (PMC10902330; doi:10.1038/s42003-024-05894-3)
Supplement: Supplementary file 2 — Description of Additional Supplementary Files [file 42003_2024_5894_MOESM2_ESM.pdf]

## Description of Additional Supplementary Files

**File name:** Supplemental Data 1
**Description:** Result of BioCycle (and JTK_CYCLE) analysis of Linden et al., 2010

**File name:** Supplemental Data 2
**Description:** Result of BioCycle analysis of Hendricks et al., 2014

**File name:** Supplemental Data 3
**Description:** Result of BioCycle analysis of our RNA-seq data.

**File name:** Supplemental Data 4
**Description:** Putative artifact in Linden et al., 2010

**File name:** Supplemental Data 5
**Description:** Source data behind the graphs in the manuscript.
